# Role of casual contact in drug-resistant tuberculosis transmission: a molecular epidemiology study

**DOI:** 10.1093/ajrccm/aamag140

**Published:** 2026-04-28

**Authors:** Neel R Gandhi, Kogieleum Naidoo, Keeren Lutchminarain, Shaheed V Omar, Hikari Yoshii, Fay Willis, Resha Boodhram, Thabisile Gwala, Angela Campbell, Megan M Coe, A Nichole Evans, Linrui Tang, Melanie H Chitwood, Senzo R Hlathi, Patience N Mbatha, Lavania Joseph, Hermina Van Der Meulen, Koleka Mlisana, Samuel M Jenness, Mark N Lurie, Barry N Kreiswirth, Joshua L Warren, Kristin N Nelson, Sara C Auld, James C M Brust, Ted Cohen, Barun Mathema, N Sarita Shah

**Affiliations:** Department of Epidemiology, Rollins School of Public Health, Emory University, Atlanta, GA, United States; Department of Medicine, School of Medicine, Emory University, Atlanta, GA, United States; CAPRISA-MRC HIV/TB Pathogenesis and Treatment Research Unit, Centre for the AIDS Programme of Research in South Africa (CAPRISA), Durban, South Africa; Department of Medical Microbiology, School of Laboratory Medicine and Medical Science, University of KwaZulu Natal; Inkosi Albert Luthuli Central Hospital, Durban, South Africa; Centre for Enteric Diseases, National Institute for Communicable Diseases, National Health Laboratory Service, Johannesburg, South Africa; Centre for Tuberculosis, National and WHO Supranational TB Reference Laboratory, National Institute for Communicable Diseases, National Health Laboratory Service, Johannesburg, South Africa; Department of Pediatrics, School of Medicine, Emory University, Atlanta, GA, United States; Department of Epidemiology, Rollins School of Public Health, Emory University, Atlanta, GA, United States; CAPRISA-MRC HIV/TB Pathogenesis and Treatment Research Unit, Centre for the AIDS Programme of Research in South Africa (CAPRISA), Durban, South Africa; Centre for Tuberculosis, National and WHO Supranational TB Reference Laboratory, National Institute for Communicable Diseases, National Health Laboratory Service, Johannesburg, South Africa; Department of Epidemiology, Rollins School of Public Health, Emory University, Atlanta, GA, United States; Department of Global Health, University of Washington, Seattle, WA, United States; Department of Epidemiology, Rollins School of Public Health, Emory University, Atlanta, GA, United States; Department of Epidemiology, Mailman School of Public Health, Columbia University, New York, NY, United States; Department of Epidemiology of Microbial Diseases, Yale School of Public Health, New Haven, CT, United States; CAPRISA-MRC HIV/TB Pathogenesis and Treatment Research Unit, Centre for the AIDS Programme of Research in South Africa (CAPRISA), Durban, South Africa; CAPRISA-MRC HIV/TB Pathogenesis and Treatment Research Unit, Centre for the AIDS Programme of Research in South Africa (CAPRISA), Durban, South Africa; Centre for Tuberculosis, National and WHO Supranational TB Reference Laboratory, National Institute for Communicable Diseases, National Health Laboratory Service, Johannesburg, South Africa; Centre for Tuberculosis, National and WHO Supranational TB Reference Laboratory, National Institute for Communicable Diseases, National Health Laboratory Service, Johannesburg, South Africa; Department of Medical Microbiology, School of Laboratory Medicine and Medical Science, University of KwaZulu Natal; Inkosi Albert Luthuli Central Hospital, Durban, South Africa; National Health and Laboratory Service, Johannesburg, South Africa; Department of Epidemiology, Rollins School of Public Health, Emory University, Atlanta, GA, United States; Department of Epidemiology, School of Public Health, Brown University, Providence, RI, United States; Center for Discovery and Innovation, Hackensack Meridien Health, Nutley, NJ, United States; Department of Biostatistics, Yale School of Public Health, New Haven, CT, United States; Department of Epidemiology, Rollins School of Public Health, Emory University, Atlanta, GA, United States; Department of Epidemiology, Rollins School of Public Health, Emory University, Atlanta, GA, United States; Department of Medicine, School of Medicine, Emory University, Atlanta, GA, United States; Department of Medicine, Albert Einstein College of Medicine and Montefiore Medical Center, Bronx, NY, United States; Department of Epidemiology of Microbial Diseases, Yale School of Public Health, New Haven, CT, United States; Department of Epidemiology, Mailman School of Public Health, Columbia University, New York, NY, United States; Department of Epidemiology, Rollins School of Public Health, Emory University, Atlanta, GA, United States; Department of Medicine, School of Medicine, Emory University, Atlanta, GA, United States

**Keywords:** tuberculosis, transmission, whole genome sequencing, drug resistance

## Abstract

**Rationale:**

Transmission is the primary driver of tuberculosis (TB) and drug-resistant TB (DR-TB) in high-burden countries; however, where and between whom spread occurs is poorly understood.

**Objectives:**

We conducted universal whole genome sequencing (WGS) to evaluate the role of casual contact in *Mycobacterium tuberculosis* transmission.

**Methods:**

We recruited persons diagnosed with second-line DR-TB (eg, extensively drug-resistant [XDR], pre-XDR-TB) from June 2018 to December 2022 in metropolitan Durban, South Africa. We collected named contacts and GPS coordinates of homes, clinics, and community locations visited regularly before diagnosis. Among participants genotypically clustered by whole genome sequencing (≤12 single-nucleotide polymorphisms), we quantified the proportion attributable to close versus casual contact. Close contact was defined as person-to-person links or overlapping hospitalizations. Casual contact links were based on geographic proximity of homes and community locations, or shared outpatient clinics.

**Measurements and Main Results:**

We enrolled 305 of 383 (80%) persons diagnosed with second-line DR-TB. TB isolates were sequenced for 251 (83%) participants; 141 (56%) were genotypically linked, forming 25 clusters (range, 2-49 persons/cluster). Among clustered participants, 69 (49%) were epidemiologically linked by casual contact and 13 (9%) through close contact. Multivariable analysis identified living within 1 km (OR, 17.9), visiting proximate community locations (OR, 1.88), shared outpatient clinic (OR, 1.72), and person-to-person links (OR, 5.38) as significant risk factors associated with genotypic clustering.

**Conclusions:**

Casual contact in community locations accounted for half of transmission among genotypic clusters in a high-burden setting. Efforts to curb TB will require a greater emphasis on community-based measures to identify cases from casual contact or undetected intermediate cases, in addition to the current mainstay of contact tracing.

At a Glance Commentary **Scientific Knowledge on the Subject:** Tuberculosis (TB) transmission is traditionally thought to occur due to close and prolonged contact. However, among genotypically clustered TB patients in population-based studies, <30% are found to have an epidemiologic link through close contact. Transmission through casual contact is hypothesized to drive TB incidence in high-burden settings, but few data are available to confirm this hypothesis.

**What This Study Adds to the Field:** The CONTEXT study used universal whole genome sequencing (WGS), geospatial, and epidemiologic analysis among patients with drug-resistant TB to quantify the proportion of transmission that occurs through casual contact vs close contact. We found that only 9% of WGS-clustered participants had an epidemiologic link through close contact. Addition of casual contact to the analysis identified an additional 49% of epidemiologic links, confirming the important role of casual contact in TB transmission in high-burden settings. Efforts to curb TB transmission in high-burden settings will need to go beyond tracing of only close contacts in homes and congregate settings. Broader, community-based approaches to screening and transmission prevention are needed to reduce TB incidence and achieve the WHO End TB goals.

Impact of this Research

The CONTEXT study utilized universal whole genome sequencing, geospatial, and epidemiologic analysis to quantify the proportion of *Mycobacterium tuberculosis* transmission that occurs through casual contact versus close contact. We found that while only 9% of WGS-clustered participants had an epidemiologic link through close contact, an additional 49% of epidemiologic links were found through casual contact, confirming the important role of casual contact in *Mtb* transmission in high-burden settings. These findings suggest that efforts to curb the TB epidemic in high-burden settings will need to go beyond contact tracing of only close contacts of TB patients. Broader, community-based approaches to screening and transmission prevention will be needed to reduce TB incidence and achieve the WHO End TB goals by 2035.

## Introduction

Tuberculosis (TB) remains the leading infectious cause of death worldwide.^1^ Despite progress in reducing TB incidence and deaths, the emergence of multidrug-resistant (MDR) and extensively drug-resistant (XDR) TB has undermined these gains. Treatment of MDR-TB and XDR-TB requires use of second-line TB medications that are more toxic and less effective than first-line drugs. Although new regimens containing bedaquiline and linezolid hold promise, access is limited and resistance to both drugs has already emerged.^2^^,^^3^ Therefore, strategies to prevent MDR- and XDR-TB remain paramount.

The majority of MDR- and XDR-TB in high-incidence settings occurs through transmission of drug-resistant *Mycobacterium tuberculosis* (*Mtb*) strains.^4^^,^^5^ However, interrupting *Mtb* transmission remains a persistent challenge due to an inability to identify where and between whom the majority of transmission is occurring. *Mtb* transmission is generally thought to occur when there is prolonged, close contact—conditions typically seen in households or congregate settings^6^—however, studies have shown that, even with intensive contact investigation, only 9% to 30% of genotypically linked TB cases may be attributed to close contact.^4^^,^^7-9^ This suggests that a large portion of TB is transmitted outside households and congregate settings, through more limited, casual contact in community settings. For example, a meta-analysis of ∼14 000 child TB contacts found that <20% of transmission was attributable to household exposure.^10^ Attributing transmission to community settings is challenging, however, because TB index patients are not likely to name individuals with whom they had only brief and low-intensity interactions as part of everyday activities. Further, given the airborne nature of *Mtb* transmission, infectious patients may not be aware of all individuals with whom they shared airspace (eg, in a store, church) and to whom transmission may have occurred.^11^ Although numerous studies have hypothesized that the majority of *Mtb* transmission may be occurring through casual contact in community settings, this has not been validated using robust genomic and epidemiologic methods.^12^^,^^13^

Genotyping and whole genome sequencing (WGS) are essential tools for characterizing *Mtb* transmission^14^ but have been underutilized in high-burden countries due to cost and limited laboratory infrastructure. Thus, transmission dynamics in high-burden countries are not well understood. “Universal,” population-based genotyping has been implemented in the United States and Europe over the past 2 decades and has uncovered previously unknown routes of *Mtb* transmission among individuals who do not name each other as contacts.^15^^,^^16^ Many of these individuals have subsequently been found to have common social networks or shared community locations (eg, restaurants, bars).^11^^,^^17-19^ However, TB prevention efforts remain primarily focused on investigation of close contacts, particularly those within households. If casual contact is the dominant mode of *Mtb* transmission, this would have important implications for global TB prevention programs and the design of more effective preventive interventions.^20^

South Africa has an estimated 270 000 new TB cases and 13 000 MDR-TB cases annually,^1^ despite substantial investments in TB diagnostics and therapeutics and improvements in HIV care. To understand the contribution of casual contact as a driver of TB incidence, we conducted the CONTEXT study, a cross-sectional, universal WGS study of patients with second-line drug resistance (eg, pre-XDR, XDR-TB) in KwaZulu-Natal province, South Africa. Some of the results of this study have been previously reported in the form of an abstract.^21^

## Methods

### Setting

KwaZulu-Natal has a single referral laboratory that conducts all second-line TB drug-susceptibility testing (DST), allowing for maximal capture of all persons diagnosed with pre-XDR-TB and XDR-TB in the province. During the study period, persons diagnosed with rifampin resistance by Xpert MTB/RIF Ultra (Cepheid, Sunnyvale, CA, United States) were recommended to have a sputum sample sent to the provincial laboratory for DST to fluoroquinolones and second-line injectables on the Genotype MTBDR*sl* assay (Hain Lifesciences, Nehren, Germany). In 2021, phenotypic DST was initiated for bedaquiline, linezolid, and clofazimine using the MGIT 960 method (each at 1 μg/mL).

The 2018 WHO definitions of pre-XDR-TB (resistance to at least isoniazid and rifampin, and either a fluoroquinolone or a second-line injectable medication) and XDR-TB (resistance to at least isoniazid, rifampin, and both a fluoroquinolone and a second-line injectable medication) were used across the full study period for consistency, despite revisions to the definitions in 2021.^22^

### Study design and data collection

We prospectively recruited all individuals diagnosed with resistance to at least one second-line TB drug (eg, fluoroquinolones, second-line injectables) from 2018 to 2022 from any health facility in the districts of eThekwini, iLembe, Ugu, or uMgungundlovu of KwaZulu-Natal province (total population 7.0 million, land area 20 218 km^2^; Figure S1).

Participants underwent structured interviews to collect information from the 2 years before enrollment regarding (1) sociodemographics, medical history, and clinical characteristics; (2) home residence(s); (3) community locations where they spent ≥2 hours/week most weeks; (4) daily movement and transportation use; (5) places they visited for ≥5 cumulative nights for any reason (eg, work, family); (6) inpatient hospital admissions for any diagnosis; and (7) outpatient clinics attended for any reason (eg, TB, prenatal care, primary care). GPS coordinates were collected at all locations and participants were asked to list all individuals with whom they had close contact (ie, touching, talking with, or spending time near) at each location. (see Supplementary Methods for detailed description of data collection).

For each participant, the diagnostic *Mtb* isolate was regrown and shipped to the National Institute for Communicable Diseases in Johannesburg, South Africa, for WGS.

### Epidemiologic analyses and definitions

*Close contact links* were defined as person-to-person links or overlapping hospitalizations (see Supplementary Methods for full details):

- Person-to-person link: Participant who named another study participant(s) as a close contact or 2 participants who named the same close contact. Individuals were matched by first and last name using modified Levenshtein string distance ≤3 as a cutoff^23^; age and sex were also considered, with validation performed by 2 native Zulu-speaking research staff.
- Overlapping hospitalization: Participants admitted to the same hospital when one was infectious (30 days before, to any time after, TB diagnosis) and the other was in the vulnerable period (>30 days prior to diagnosis) to become infected. We also conducted sensitivity analysis using 60 days and 90 days for the definitions of infectious and vulnerable periods.

For participants who did not have a close contact link, we examined *casual contact links* to identify participants who may have had casual interactions with each other in their daily lives based on living geographically close to one another (residential proximity), frequently visiting a community location geographically close to another participant (community proximity), or attending the same outpatient clinic:

- Outpatient clinic link: Participants who attended the same outpatient clinic for any reason (eg, TB, HIV, maternity, primary care).
- Residential proximity link was defined as a 1 km distance, based on GPS coordinates, between participants’ home residences for the primary analysis.
- Community proximity link was defined as a ≤500-meter distance between a participant’s named community location and another participant’s home or named community location for the primary analysis.

A shorter radius was used for community proximity than residential proximity to acknowledge that participants’ activity space around community locations (eg, work, school, friend’s house) may be narrower than in their residential neighborhoods. For both proximity measures, we also carried out sensitivity analyses varying the distances from 250 meters to 2 km to assess the impact of differing distances.

### Whole genome sequencing and genotypic clustering

Participants’ DR-TB isolates were regrown and DNA extracted for WGS on the Illumina MiSeq platform (Illumina, San Diego, CA, United States; see Supplementary Methods for detailed genotyping methods). All isolates had reads covering >99% of the reference genome and the lowest mean coverage depth for any isolate was 15×. More than 93% of isolates have mean coverage depth over 50×.

Single-nucleotide polymorphisms (SNPs) were detected using standard pairwise resequencing techniques. Pairwise SNP differences were calculated for all participants. Although there is no current consensus for an optimal pairwise SNP difference to indicate transmission, higher SNP thresholds, such as ≤10 or ≤12 SNPs, have been used in high-burden settings where analyses of within-host diversity and close contacts have demonstrated that a higher threshold may be needed to capture transmission.^5^^,^^7^ In this study, we defined clustering among participants with ≤12 SNPs, but also explored additional thresholds (eg, ≤5 SNPs, ≤20 SNPs).

### Integrated epidemiologic and genomic analysis

We combined epidemiologic, genomic, and geospatial data to infer transmission. Among genotypically clustered participants (≤12 SNPs), we determined if epidemiologic links existed and, if so, the type of link. We first examined close contact links, if participants had more than one epidemiologic link. Among those with only casual contact links, we described whether pairs had one or more types of links (eg, residential proximity, community proximity).

### Multivariable analysis of genotypic clustering

We performed an epidemiological analysis to estimate the association between casual contact and genotypic clustering. We fit a hierarchical Bayesian dyadic regression model with a binary outcome (individuals with sequences ≤12 SNPs apart [outcome = 1] vs >12 SNPs [outcome = 0]) among individuals with both WGS and home GPS data available. We included the various epidemiological links, as well as sex, age, and HIV status in the model to estimate their associations with genotypic clustering. We used the R package GenePair,^24^ which includes spatially structured individual-level random effect parameters to account for multiple sources of correlation (ie, network dependence and spatial) across dyadic outcomes, resulting in more robust statistical inference than that obtained from methods that ignore correlation (see Supplementary Methods for a full model description, details about model convergence, and sensitivity analyses for several of the model assumptions).

### Ethical considerations

Informed consent was obtained from all study participants (or next-of-kin, if deceased or too ill to provide consent). The ethics committees at the University of KwaZulu-Natal and Emory University approved the study.

## Results

From June 2018 through December 2022, 383 persons were diagnosed with TB with second-line drug resistance (eg, pre-XDR or XDR-TB) within the study catchment area and were eligible for enrollment. We consented and completed interviews for 305 participants (80%; Supplementary Figure S2). Enrolled individuals did not differ significantly in age, sex, or drug-susceptibility pattern from those not enrolled (*P* = .23, .31, and .47, respectively; Table S1).

Among interviewed participants, 137 (45%) were female and the median age was 37 years (IQR, 30-44 years) (Table 1). There were 216 (73%) participants with HIV coinfection: 203 (96%) were receiving antiretroviral treatment, median CD4 count was 363 cells/μL (IQR, 182-621 cells/μL), and 86 (59%) had a viral load of <20 copies/mL at enrollment. The drug resistance category was XDR-TB for 100 (33%) participants and pre-XDR-TB for 170 (56%).

**Table 1:** Baseline demographic, risk factor, and clinical characteristics of study participants.

Characteristic	Study cohort (*N* = 305)	
	***N*** ^a^	**%**
**Sex**		
**Female**	137	45%
**Male**	168	55%
**Age, y, median (IQR)**	37 (30-44)	
**0-12**	1	0.3%
**13-19**	12	3.9%
**20-29**	61	20%
**30-39**	117	38%
**40-49**	70	23%
**≥50**	44	14%
**Employment**		
**Currently employed or employed immediately before TB illness**	85	29%
**Employed in the last 2 y**	35	12%
**Not employed in the last 2 y**	172	59%
**Education (highest level completed)**		
**No formal schooling**	8	2.6%
**Primary school**	43	14%
**Some secondary school**	166	55%
**Graduated secondary school**	69	23%
**University or other higher degree**	16	5.3%
**TB risk group**		
**Healthcare worker**	15	4.9%
**Incarcerated in last 12 mo**	10	3.3%
**Prior mine worker**	5	1.6%
**Alcohol use frequency**		
**Never**	183	61%
**4 times a month or less**	94	31%
**≥2 times a week**	22	7.3%
**Smoked at least 100 cigarettes in lifetime**		
**Yes**	112	37%
**No**	191	63%
**TB and HIV disease characteristics**		
**Cough in past 6 mo**		
**Yes**	154	51%
** No**	146	49%
** Previous DR-TB diagnosis**		
**Yes**	31	13%
**No**	202	87%
**Resistance pattern**		
**XDR-TB**	100	33%
**Pre-XDR (fluoroquinolone-resistant)**	130	43%
**Pre-XDR (injectable-resistant)**	40	13%
**MDR with other SL resistance**	9	3.0%
**SL resistance, but susceptible to INH and/or RIF**	26	8.5%
**HIV status**		
**Positive**	216	73%
**Negative**	78	27%
**On ART (at time of enrollment)** ^b^		
**Yes**	203	96%
**No**	9	4.2%
** CD4 count (at time of enrollment), cells/μL, median (IQR)** ^b^	363 (182-621)	
≤**50**	6	4.0%
**51-200**	37	25%
**201-350**	27	18%
**351-500**	26	17%
**>500**	55	36%
**Viral load (at time of enrollment), copies/mL** ^b^		
**<20**	86	59%
**20-1000**	23	16%
**1001-10** **000**	14	9.7%
**10** **001-100** **000**	11	7.6%
**>100** **000**	11	7.6%

Abbreviations: ART, antiretroviral therapy; DR, drug resistant; INH, isoniazid; IQR, interquartile range; MDR, multidrug resistant; RIF, rifampin; SL, second line; TB, tuberculosis; XDR, extensively drug resistant.

a When the sum of a column for a variable is less than *N* = 305, the difference represents participants for whom that variable’s result was missing or unknown.

b Among the *n* = 216 participants who were HIV positive.

### Close contacts and potential locations for *Mtb* transmission

Participants named 2929 close contacts (median, 9 [IQR, 6-13] contacts per participant). The most common places of interaction were at their own home (*n* = 1402 [48%]), friend/family member’s home (*n* = 556 [19%]), or a place where they visited overnight (*n* = 256 [8.7%]) (Table S2).

There were 218 (71%) participants who reported a hospitalization; 80 (37%) were hospitalized at more than one hospital. Of these, 50 (23%) were admitted during the vulnerable period before DR-TB diagnosis.

Participants named 605 unique locations where they spent ≥ 2 hours in a typical week (total locations named: *n* = 721; median, 3 [IQR, 2-3] locations per participant). These locations were most commonly friend/family member’s home (*n* = 220 [31%]), store or shopping mall (*n* = 216 [30%]), entertainment venue (*n* = 84 [12%]), or religious place (*n* = 50 [6.9%]). Participants named 668 outpatient clinics they attended, representing 192 unique clinics (median, 2 [IQR, 2-3] per participant,). The outpatient departments most commonly visited were TB (*n* = 451 [68%]), pharmacy (*n* = 384 [57%]), outpatient or urgent care (*n* = 301 [45%]), and HIV (*n* = 292 [44%]) (Table S3).

### Whole genome sequencing and genotypic clustering

**Figure 1:**
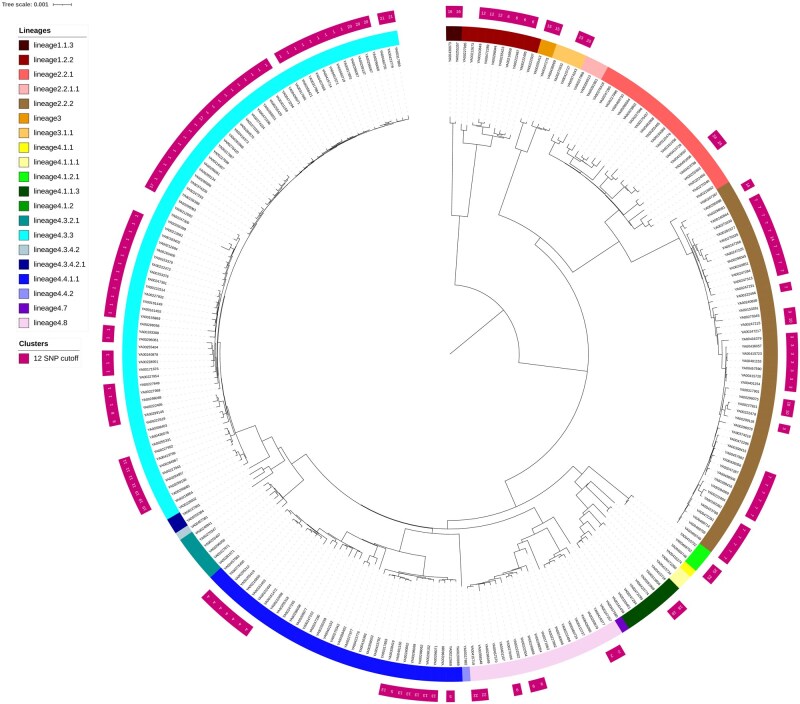
Phylogenetic tree of *Mycobacterium tuberculosis* (*Mtb*) isolates from participants who had whole genome sequencing performed (*n* = 251): *Mtb* strains from lineages 1 through 4 were found (lineage 1: *n* = 12 [4.8%], lineage 2: *n* = 74 [29%], lineage 3: *n* = 6 [2.4%], lineage 4: *n* = 159 [63%]). There were 141 (56%) isolates that were genotypically clustered with at least one other isolate at a ≤12 single-nucleotide polymorphism (SNP) threshold. A high proportion of clustering was seen within lineages 2.2.2 and 4.3.3.

WGS was successfully completed for 251 (83%) participants (Figure 1), of whom 141 (56%) had an *Mtb* isolate genotypically clustered with at least one other participant at a ≤12 SNP threshold. The matching isolates formed 25 clusters, ranging in size from 2 to 49 participants (Figure S3). The median pairwise SNP difference was 6 SNPs (IQR, 3-8) to the closest participant within a cluster. Using alternate thresholds of ≤5 and ≤20 SNPs, the number of genotypically clustered participants was 70 (28%) and 185 (65%), respectively, and the number of clusters was 20 (range, 2-7 participants per cluster) and 25 (range, 2-62 participants per cluster).

### Combined WGS and epidemiologic analysis

**Figure 2:**
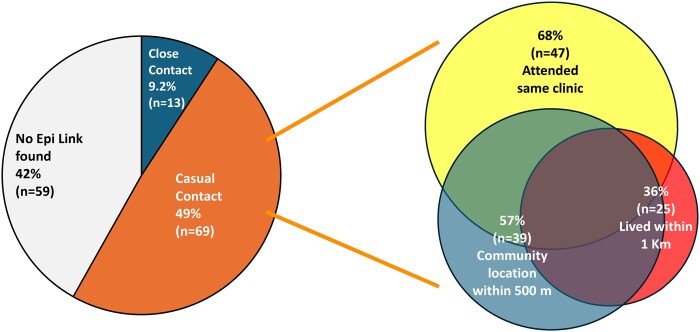
Close and casual contact epidemiologic (Epi) links among the 141 participants who were genotypically clustered at ≤12 SNP threshold: among 141 participants, 49% (*n* = 69) are epidemiologically linked by casual contact, 9.2% (*n* = 13) are linked by close contact, and 42% (*n* = 59) had no epidemiologic link found. Among those with casual contact links: 36% (*n* = 25) lived within 1 km of another participant (residential proximity link), 57% (*n* = 39) regularly visited a community location within 500 meters of another participant’s home or community location (community proximity link), and 68% (*n* = 47) attended the same outpatient clinic. There were 31 (45%) participants who had more than one type of casual contact link.

Among 141 participants genotypically clustered at ≤12 SNPs, 82 (58%) could be linked *epidemiologically* to at least one other participant in their cluster (Figure 2): 13 (9.2%) were linked through *close contact* and an additional 69 (49%) were linked through *casual contact*. All 13 participants linked by close contact had person-to-person links; none had hospital links.

**Figure 3:**
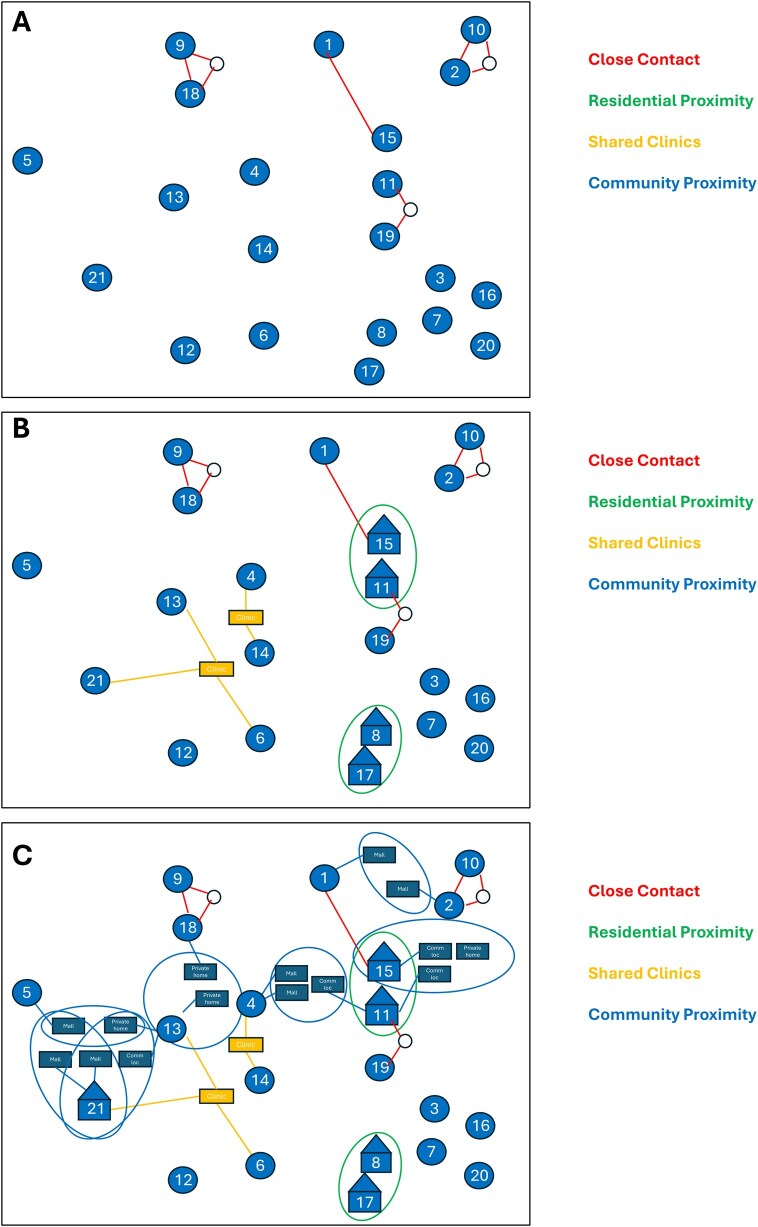
Casual contact links provide additional epidemiologic connections between genotypically clustered individuals: Example from a genotypic cluster of 21 participants: Inclusion of casual contact links increases the number of clustered participants with an epidemiologic link from 8 (38%) to 16 (76%) in this genotypic cluster (blue numbered circles represent participants in this genotypic cluster). (A) Eight participants are epidemiologically connected through close contact (red lines indicate person-to-person links; small white circles represent shared named contacts). (B) The inclusion of residential proximity links (green ovals), and shared clinic links (yellow lines connecting) identifies epidemiologic links for an additional 7 participants within the genotypic cluster (yellow rectangles represent outpatient clinics). (C) The addition of community proximity links (blue ovals connecting homes and/or blue rectangles representing community locations) identify epidemiologic links for an additional participant in the genotypic cluster.

Among the 69 participants linked through *casual contact*, 25 (36%) lived within 1 km of a genotypically clustered participant (residential proximity link), 39 (57%) regularly visited a communitylocation within 500 meters of another participant’s home or community location (community proximity link), and 47 (68%) attended the same outpatient clinic (Figures 2 and 3). There were 31 (45%) participants with more than one type of casual contact link. We did not identify an epidemiologic link for 59 (42%) genotypically clustered participants.

The relative proportion of clustered participants who had epidemiologic links through casual contact versus close contact remained similar when using ≤5 and ≤20 SNP thresholds (Table S4). Similarly, there were no differences in the median SNP difference between pairs who had casual contact versus close contact (Table S5).

### Multivariable analysis of genotypic clustering

**Figure 4:**
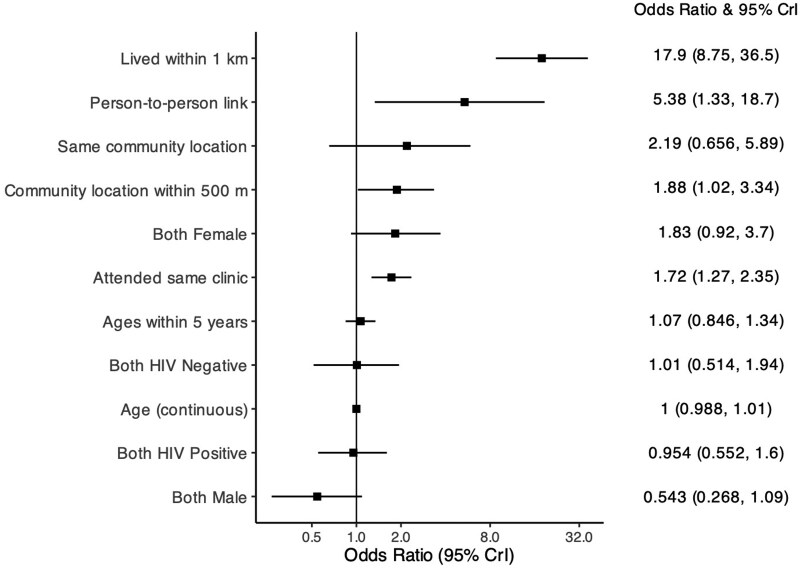
Multivariable GenePair analysis of genotypic clustering at ≤12 single-nucleotide polymorphism threshold. Four factors were associated with genotypic clustering with another participant: participants who lived within 1 km of each other (residential proximity link); named each other, or the same individual, as a close contact (person-to-person link); visited a community location within 500 meters of the other’s home or community location (community proximity link); or attended the same outpatient clinic. Age, sex, and HIV status were not associated with genotypic clustering. Abbreviation: CrI, credible interval.

The multivariable Bayesian analysis (GenePair) identified residential proximity, shared clinic, community proximity, and person-to-person links as important risk factors associated with genotypic clustering (Figure 4). Living within 1 km of each other (residential proximity) had the strongest estimated association (odds ratio [OR], 17.9 [95% credible interval {CrI}, 8.8-36.5]). Pairs with a person-to-person link (OR, 5.38 [95% CrI, 1.33-18.7]), community proximity link (OR, 1.88 [95% CrI, 1.02-3.34]), and shared outpatient clinic (OR, 1.73 [95% CrI, 1.27-2.35]) also had an increased odds of genetic clustering (Figure 4).

## Discussion

The global TB epidemic is being driven by airborne transmission, but less than one-third of transmission can be explained by close contact. We combined universal WGS, epidemiologic, and geospatial analyses to characterize the contribution of casual contact to transmission of drug-resistant TB in an endemic setting. We identified casual contact epidemiologic links for 49% of genotypically clustered participants, beyond those who could be connected through close contact. Proximity of home residence or regularly visited community locations, in addition to shared outpatient clinics, were associated with clustering. These findings provide rigorous new data challenging the long-standing belief that public health interventions in homes and congregate settings would be sufficient to impact TB incidence in high-burden settings. Instead, these data support the notion that the predominance of *Mtb* transmission is occurring through more limited, casual contact. Efforts to meaningfully reduce TB incidence in high-burden settings will require initiatives that focus on curbing *Mtb* transmission at a community level or the advent of a new, mass prevention tool, such as a TB vaccine.

As TB genotyping methods have become more precise and more widely utilized, a consistent finding has been that only a minority of genotypically clustered individuals, particularly in high-burden countries, have an epidemiologic link identified through close contact.^7-9^ Household contact studies in high-burden settings have similarly found that only a minority of secondary cases can be genotypically linked to their index cases, suggesting that a large portion of TB is transmitted outside households, perhaps through more limited, casual contact in community settings.^9^^,^^25^ However, studies to date have not confirmed this hypothesis, in part due to the difficulty in designing a study that links individuals who do not name each other and may not know that they have shared the same airspace. Indeed, data suggest *Mtb* bacilli can remain airborne for several hours, making it possible for transmission to occur between 2 individuals even if they are not in the same space at the same time.^26^^,^^27^ The number of community contacts an individual has is larger than the number of household contacts, creating greater opportunity for exposure and infection outside the home, despite generally lower intensity of interaction.^28^

A major strength of our study is the rigorous design that utilized a combination of WGS, epidemiologic investigation, and geospatial analysis to overcome the challenges of characterizing *Mtb* transmission by casual contact. Specifically, universal WGS and phylogenetic methods allowed us to identify individuals between whom transmission is likely to have occurred, irrespective of whether they named each other. Detailed interviews and geospatial analysis of homes and regularly visited community locations provided epidemiologic data to link individuals with a high likelihood to have shared air space. Integration of these methods provided evidence that casual contact accounts for a substantial proportion of transmission events not explained by close contact. Multivariable analysis further supported the role of residential proximity, community locations proximity, and shared outpatient clinics in casual contact transmission links.

Community transmission may be driven in part by individuals with subclinical or asymptomatic TB who are unaware of their disease status, but capable of transmitting.^29^ During this time, they may continue their normal work or social activities while unknowingly infecting others. In our study, we found that only 50% of all participants, including only 50% of clustered participants (70 of 141), reported cough in the 6 months before diagnosis. Efforts to increase community-based screening, such as mass community-based chest X-ray or molecular diagnostic testing campaigns, will likely be needed to meaningfully impact *Mtb* transmission by casual contact.^30^^,^^31^ Additional interventions, such as improving ventilation in public settings (eg, public transportation, schools, shopping malls), educational campaigns to prompt earlier care seeking for TB diagnosis, and poverty alleviation measures that address underlying social determinants are critical. Our study population reflects a high social vulnerability based on low access to heating, piped water, and flush toilets, and high unemployment and household crowding.^32^

Our study focused on individuals who had DR-TB, given the importance of maximal capture in transmission studies to identify potential transmission links. By focusing on patients with pre-XDR and XDR-TB, it was feasible to enroll the vast majority of individuals diagnosed. Similar transmission studies of drug-susceptible TB (DS-TB) are near impossible to carry out in high-TB-burden settings due to the overwhelming caseload in a given geographicarea in a discrete time period (>100 000 DS-TB diagnoses in KwaZulu-Natal during the study period). While differences may exist in the clinical presentations or other behavioral patterns between individuals with DS-TB or DR-TB, our finding that half of DR-TB transmission may be attributable to casual contact may provide valuable insights for extrapolation to the wider TB epidemic in high-burden settings.

Our study has limitations. Although we sought to enroll and sequence all persons with second-line DR-TB, our analysis is limited to those who were diagnosed with culture-confirmed DR-TB disease. There may be individuals who were part of transmission chains who had asymptomatic TB, did not seek medical care, or did not have DST done (eg, missed diagnosis, lost sputum sample). In addition, individuals who developed latent *Mtb* infection because of DR-TB transmission but who did not progress to culture-positive disease are not included. Migration into and out of the study catchment area and sampling only over the 4-year study period also may have led to individuals who were not included in the study. Taken together, these factors would result in an underestimate of the proportion of clustered cases and the number of epidemiologic links. Second, our study did not collect every location a participant visited in the 2 years before diagnosis. We focused on the most commonly visited locations as repeated presence at those locations increases the likelihood of transmission occurring there. Collecting more locations would have increased the likelihood of identifying a casual contact link; thus, our findings are likely a minimum estimate of transmission through casual contact. Third, data collected through interviews are subject to recall bias. Recall bias can be minimized by eliciting information over a short period prior to the interview; however, given the variable incubation period in TB and that greatest likelihood of progression is in the first 2 years following infection, we believed it was important to elicit information about homes, community locations, and hospitalizations over the 2 years prior to diagnosis. Finally, the study was designed as a cross-sectional study at the time of TB diagnosis. This limited our ability to assess activities and movement in the period after diagnosis and before culture conversion.

Substantially reducing TB incidence will not be possible without new strategies for halting *Mtb* transmission in high-burden countries. Our study demonstrates that casual contact in community settings may explain half or more of all transmission in this high-burden setting. These findings support the long-hypothesized notion that transmission by casual contact plays a predominant role in high-burden settings, suggesting that efforts to reduce TB incidence will require community-level interventions that go beyond the current mainstay of contact investigation.

## Supplementary Material

aamag140_Supplementary_Data

## Data Availability

This article has a data supplementary, which is accessible at the Supplements tab.
