# Supplementary material for: Role of casual contact in drug-resistant tuberculosis transmission: a molecular epidemiology study
Source: Am J Respir Crit Care Med. 2026 Apr 28;212(7):1585–95. doi: 10.1093/ajrccm/aamag140 (PMC13318217; doi:10.1093/ajrccm/aamag140)
Supplement: aamag140_Supplementary_Data [file aamag140_supplementary_data.zip › aamag140_Supplementary_Data/Supplement 1-6-26 clean.docx]

**ONLINE DATA SUPPLEMENT**

**Role of Casual Contact in Drug-Resistant Tuberculosis Transmission: A Molecular Epidemiology Study**

Neel R. Gandhi, MD*; Kogieleum Naidoo, PhD*, PhD; Keeren Lutchminarain, MBChB; Shaheed V. Omar, PhD; Hikari Yoshii, MD; Fay Willis, MPH; Resha Boodhram; Thabisile Gwala; Angela Campbell, MS; Megan M. Coe, PhD; A. Nichole Evans, MS; Linrui Tang, MA; Melanie Chitwood, PhD; Senzo R. Hlathi; Patience N. Mbatha; Lavania Joseph MSc; Hermina Van Der Meulen; Koleka Mlisana, PhD; Samuel M Jenness, PhD; Mark N. Lurie, PhD; Barry N. Kreiswirth, PhD; Joshua L. Warren, PhD; Kristin N. Nelson, PhD; Sara C. Auld, MD; James C.M. Brust, MD; Ted Cohen, DPH; Barun Mathema, PhD^8^; N. Sarita Shah, MD

**Table of Contents**

[Supplementary Methods 2](#_Toc218523055)

[Data Collection 2](#_Toc218523056)

[Selection of *Mtb* isolate for WGS 5](#_Toc218523057)

[Identification of epidemiologic links 6](#_Toc218523058)

[Whole genome sequencing (WGS) methods 9](#_Toc218523059)

[Multivariable GenePair analysis 10](#_Toc218523060)

[Supplemental Figures 13](#_Toc218523061)

[Supplemental Figure E1: Map of geographic catchment area of the study within KwaZulu-Natal province, South Africa 13](#_Toc218523062)

[Supplemental Figure E2: Enrollment flow diagram 14](#_Toc218523063)

[Supplemental Figure E3: Visualization of 25 genotypic clusters among participants linked at ≤12 SNP threshold 15](#_Toc218523064)

[Supplementary Tables 16](#_Toc218523065)

[Supplemental Table E1: Characteristics of enrolled study participants vs. non-enrolled persons with pre-XDR and XDR-TB 16](#_Toc218523066)

[Supplemental Table E2: Locations of interactions with Close Contacts 18](#_Toc218523067)

[Supplemental Table E3: Departments visited during outpatient clinic visits 19](#_Toc218523068)

[Supplemental Table E4: Sensitivity analysis of genotypic clustering and epidemiologic links using varying SNP thresholds. 20](#_Toc218523069)

[Supplemental Table E5: SNP differences among pairs with differing types of epidemiologic links. 21](#_Toc218523070)

# ****Supplementary Methods****

## Data Collection

Study eligibility and catchment area

The standard of care in KwaZulu-Natal province at the time of study initiation in 2018 was that all individuals found to have rifampin resistance by Xpert MTB/RIF Ultra (Cepheid, Sunnyvale, CA, USA) were recommended to have a second sample sent to the provincial TB referral laboratory for DST to fluoroquinolones and second-line injectables on the Genotype MTBDR*sl* assay (Hain Lifesciences, Nehren, Germany). This second sample was also tested for DST to isoniazid and rifampin on the Genotype MTBDR*plus* assay (Hain Lifesciences, Nehren, Germany).

Individuals of who were diagnosed with resistance to at least one fluoroquinolone and/or second-line injectable medication were eligible for study enrollment. Eligibility criteria included individuals of all ages who were diagnosed at a healthcare facility within the study catchment area (eThekwini, iLembe, Ugu, or uMgungundlovu districts of KwaZulu-Natal province). The map in **Supplemental Figure E1** shows the location of eligible health care facilities.

Identification of eligible participants and enrollment

On a weekly basis, laboratory results of *Mtb* isolates that were resistant to fluoroquinolones or second-line injectables were identified by the provincial TB referral laboratory for potential enrollment into the study. A study staff member then contacted the health facility from which the sample was sent to request referral of the patient for study screening. All interested patients (or their next of kin, if the patient had died or was too ill to provide consent) were contacted by study staff who explained the study and obtained written informed consent prior to initiating the data collection.

Study interview

Consented participants underwent a detailed interview to collect information about the following:

**Demographics**: age, sex, education, marital status, employment, birthplace, and residence.

**Medical history**: current and past symptoms related to TB, risk factors for TB (healthcare worker, current/former mine worker, history of incarceration), HIV status and antiretroviral treatment, comorbid conditions (diabetes, cancer), alcohol and tobacco use, hospitalizations in past 2 years, and outpatient clinics visited.

**Residential history**: all locations the participant lived for 1 month or more in the 2 years before enrollment.

**Community locations frequently visited**: all locations the participant visited “for at least 2 hours most weeks” in the 2 years before enrollment. Study staff used structured questionnaires to prompt recall of locations participants visited for work, school, religious purposes, family or close friends, shopping, entertainment (e.g., nightclubs, drinking, movies), hair salons, or gym.

**Overnight visits**: all places where the participant spent a cumulative of 5 nights or more in the 2 years before enrollment. In each location, participants were asked the reason for visiting this location (e.g., for work/school, visit family, intimate partner, illness, leisure).

Interviews were conducted in person. However, with the onset of travel restrictions in 2020 during the COVID-19 pandemic, the study protocol was amended to allow interviews to take place by phone, if needed.

Identification of household and close contacts

For each residence, participants were asked to name all household contacts. Household contacts were defined as any individual who stayed at that residence for at least 1 month in the 2 years before enrollment.

For each community and overnight visit location, participants were asked to name the people that they interacted most closely with at that location, defined as anyone they “touched, talked to or spent time near.”

For every individual named, we collected first name, last name, nickname, additional names, relationship with the participant, gender, and age.

Collection of GPS coordinates

Study staff collected GPS coordinates (exact latitude and longitude) for each residence, community location, and overnight visit location elicited within the study catchment area. In situations where it was unsafe or not possible to visit the location, or if a location was a well-known location (e.g., large shopping mall), study staff had the option to collect the latitude and longitude from a “desktop data source.” These sources included computer mapping programs (e.g., google maps).

CD4 and viral load

All participants who completed an in-person interview and were known or found to be HIV-positive had a blood sample collected for CD4 cell count and viral load measurement. For those in whom the interview was conducted by phone, CD4 and viral load data were abstracted from the South Africa Electronic Drug-Resistant Tuberculosis Register (EDRweb), a national reporting database of routinely-collected patient-level information. Results reported in Table 1 reflect a combination of results obtained from blood sample collection at enrollment and retrospective data abstraction from EDRWeb.

## Selection of *Mtb* isolate for WGS

Study staff within the KwaZulu-Natal province referral laboratory attempted to obtain the diagnostic *Mtb* isolate that first detected pre-XDR or XDR-TB for each study participant. If the diagnostic isolate was not available (e.g., due to sample loss or contamination), an alternate isolate was obtained from a sample collected within 30 days before or 90 days after the diagnostic sample.

## Identification of epidemiologic links

Person-to-person epidemiologic links

To identify person-to-person epidemiologic links, we matched named individuals using first name, last name, age, and gender. We employed modified Levenshtein distance to perform name matching (1). We used a string distance of ≤3 as a cutoff to matched named individuals. We performed further validation by having two native Zulu-speaking research staff members assign a likelihood score between 1 (identical names) and 4 (completely different names) to each pair.

Person-to-person epidemiologic links were established directly, when one participant named another participant as a contact, or when two participants named the same individual as a contact.

Overlapping hospitalizations

We asked all participants to list any time they were admitted to a hospital overnight in the past 2 years. For each hospitalization, we collected the name and location of the hospital, and dates of admission and discharge.

To identify overlapping hospitalizations where TB may have been transmitted from one participant to another, we first identified any hospital where two study participants were admitted. Next, we defined whether a participant was “infectious” or “vulnerable” during the time of the hospital admission.

We defined the *infectious period* as beginning 30 days before DR-TB diagnosis (DR-TB diagnosis is the date of sample collection which eventually was found to be drug-resistant by culture and DST) to account for infectiousness which may have pre-dated when the participant sought medical care and had a diagnostic test. We defined participants as being *vulnerable* to be infected with a DR-TB strain as anytime more than 30 days before their DR-TB diagnosis.

Two participants were considered to have an overlapping hospitalization if they were both admitted to the same hospital while one was in the infectious period and the other was in the vulnerable period. We conducted sensitivity analyses using 60 days and 90 days for the definitions of infectious and vulnerable periods.

Residential proximity links

For participants who did not have a close contact link, we examined *casual contact links* to identify participants who may have had casual interactions with each other in their daily lives based on living geographically close to one another (residential proximity), frequently visiting a community location geographically close to another participant (community proximity), or attending the same outpatient clinic.

To identify residential proximity links, home GPS coordinates for all participants were compared to one another to calculate straight line distances between them. Geographic distances of 1 kilometer, 500 meters and 250 meters were identified *a priori* as distances of interest for analysis. A straight-line distance between participants’ homes of ≤1 km was utilized to categorize a participant pair as having a residential proximity link for our main analysis. We also conducted sensitivity analyses varying the distance thresholds from 500 m to 10 km. An association between residential proximity and genotypic clustering remained regardless of the threshold used.

Community proximity links

The GPS coordinates for all community and overnight visit locations listed by a participant were compared to the homes, community locations, and overnight visit locations named by all other study participants. Similar to above, geographic distances of 1 kilometer, 500 meters and 250 meters were identified *a priori* as distances of interest for analysis. All locations that were within 500 meters of each other were categorized to be community proximity links between participants in our main analysis. We performed a sensitivity analysis of varying distance thresholds and found a linear relationship between distance and epidemiologic links.

Outpatient clinic links

Participants were asked to name any outpatient healthcare facility they attended for any reason (for themselves or anyone else) in the 2 years before enrollment. Each named outpatient facility was compared to identify clinics that were attended by 2 or more participants. The exact date of the clinic visit(s) was not used to determine overlap given limitations with precise recall of visit dates. A complete list of outpatient healthcare facilities in KwaZulu-Natal province was obtained from the KwaZulu-Natal Department of Health.

Categorization if multiple epidemiologic links are present

If a participants had both close contact links (either person-to-person, or overlapping hospitalizations) and casual contact links with any genotypically clustered study participant, we categorized that participant as having close contact links in our analyses. A participant was categorized as having casual contact links only if they did not have a close contact link with another clustered participant.

For participants that had more than one type of casual contact link with clustered participants, each of casual contact link types is described. The overlap of these various types of casual contact links is described in Figure 2 of the main manuscript.

## Whole genome sequencing (WGS) methods

Participants’ diagnostic *Mtb* isolates were regrown and DNA extracted for WGS at the National TB Reference Laboratory at the Centre for Tuberculosis – National Institute for Communicable Diseases in Johannesburg, South Africa. Libraries were prepared using Nextera XT DNA kits (Illumina, San Diego, CA, USA) and paired-end WGS performed on the Illumina Miseq platform (Illumina, San Diego, CA, USA). After removing non-*Mtb* (contaminant) reads (Kraken (2)), raw paired-end sequencing reads were generated trimmed with Prinseq (v0.20.4) (3), and aligned to the H37Rv reference genome (NC_000962.3) using the BWA-MEM (v0.7.15) (4). All isolates had reads covering >99% of the reference genome and the lowest mean coverage depth for any isolate was 15X.

SNPs were detected using standard pairwise resequencing techniques (Samtools version 0.1.19) against the H37Rv reference and filtered for quality, read consensus (>75% for the alternate allele) and proximity to indels (less than 50 base-pairs from any indel). SNPs at or within 50 base-pairs of hypervariable Pro-Pro-Glu (PPE)/Pro-Glu (PE) gene families, repeat regions, mobile elements, and known drug resistance-related genes were also excluded (5). Once SNPs were scored for confidence and quality, overlaid gene annotations were used to positions SNPs as either intergenic or genic, and synonymous or nonsynonymous. A matrix of SNPs describing polymorphisms for all strains was inferred from pairwise comparison against the reference. To examine relatedness, we constructed a maximum-likelihood phylogenetic tree using iqtree (v1.6) (6) where node robustness was evaluated using 100 bootstrap pseudoreplicates. Lineage calling was performed using fast-lineage-caller (7). Resistance was defined by the presence of WHO-listed “resistance-associated” or “resistance-associated—interim” mutations for each drug (8).

## Multivariable GenePair analysis

We estimated factors associated with genotypic clustering using a Bayesian dyadic regression model. We fit a logistic regression with the binary outcome (1 if two individuals are ≤12 SNPs apart, 0 otherwise) to data on pairs of individuals with both WGS and home GPS data available (n=250, pairs=31,125). We included the various epidemiological links (e.g., person-to-person, clinic, residential proximity, community location proximity) as well as sex, age, and HIV status in the model to estimate their associations with genotypic clustering. Because we are modeling paired outcomes, the variables for sex and HIV status have three levels (both male, both female, and male-female; both HIV positive, both HIV negative, and HIV positive-HIV negative). In both cases, we use discordant pairs (male-female, HIV positive-HIV negative) as the refence category. Similarly, age is modeled in two ways: (1) a binary indicator for the difference in individual ages (≤ 5 years) and (2) the sum of individuals’ ages.

Prior to model fitting, we performed a sensitivity analysis with a standard logistic regression model to check various model assumptions. First, we tested coding the age variable as continuous or as a factor with two, three, or five levels. We found that modeling the sum of individuals’ age as a continuous variable produced the best model fit, as determined by AIC. Second, we tested whether there was collinearity among any of the epidemiological link predictors. We fit the model with only one epidemiological link type at a time (including sex, age, and HIV status); we then fit the model with all the predictors together. We found that the estimated effect sizes for each epidemiological link did not differ significantly when we fit the model to these factors together or separately, particularly with respect to statistical significance. This suggests that the level of correlation between the variables may not have been high enough to impact their estimation within the model.

We implemented the model using the R package *GenePair* (9), which includes spatially structured individual-level random effect parameters to account for multiple sources of correlation across dyadic outcomes. Specifically, the model is given as:

$$Y_{ij}| p_{ij} \sim\mathrm{Bernoulli}\left( p_{ij} \right), i=1,\ldots, n-1, j=i+1,\ldots,n,$$

$$\mathrm{logit} \left( p_{ij} \right)=\mathbf{x}_{ij}^{T}\boldsymbol{\beta} + \left( \mathbf{d}_{i}+\mathbf{d}_{j} \right)^{T}\boldsymbol{\gamma}+\theta_{i}+\theta_{j}$$

where *Y_ij_* is the observed (binary) relationship between two individuals, *p_ij_* is probability that two individuals have isolates that differ by ≤12 SNPs, **x**_ij_ is a vector pair-level predictors with corresponding regression parameters ***β***, **d***_i_* and **d***_j_* are vectors of individual-level predictors with corresponding regression parameters ***γ***, and *θ_I_* and *θ_j_* are spatially-referenced, individual-specific random effect parameters. These random effects are decomposed into two components; one that describes spatial correlation and one for non-spatial, individual-specific variability. The spatial component is modeled using a Gaussian process prior distribution with exponential correlation structure defined by the distances between the individuals while the non-spatial component is modeled using independent Gaussian random effects. Full details can be seen in reference (9).

We based inference on samples from the joint posterior distribution, removing the first 10,000 iterations prior to convergence of the model and thinning the remaining 90,000 samples by a factor of 10 to reduce posterior autocorrelation. We assessed model convergence with Geweke’s convergence diagnostic; all **β** and **γ** parameters had a z-score between -2 and 2, except for the parameters for the association between clustering and clinic links (z-score = 2.275) and clustering and age (z-score = 2.028), suggesting adequate convergence overall. All parameters had an effective sample size greater than 300 indicating enough post-converge samples were collected to conduct posterior inference. Posterior median odds ratio estimates and corresponding 95% quantile-based equal-tailed credible intervals are presented for making statistical inference.

# Supplemental Figures

Supplemental Figure E1: Map of geographic catchment area of the study within KwaZulu-Natal province, South Africa**.** The dots indicate the location of health facilities. If a patient had a sample collected at any of the shown health facilities, they were eligible for enrollment.

**
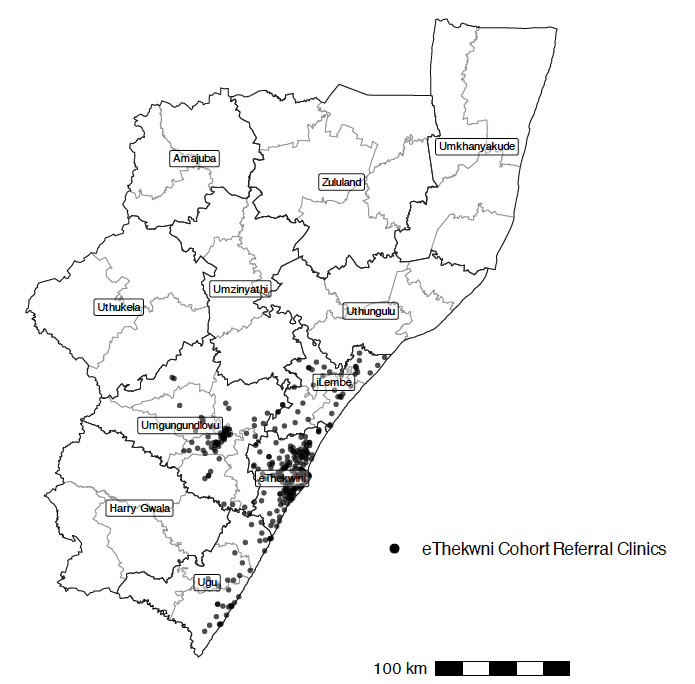
**

## Supplemental Figure E2: Enrollment flow diagram

Diagnosed within geographic catchment area

N=383

37 Could not be contacted

30 Refused participation in study

Consented for enrollment

N=316

11 Did not complete interview

Completed Interview

N=305

Supplemental Figure E3: Visualization of 25 genotypic clusters among participants linked at ≤12 SNP threshold**.** Nodes (grey circles) indicate participants’ isolates; lines connecting nodes indicate genotypic links based on a ≤12 SNP difference between participants isolates. The four largest cluster contain 49 (lineage 4.3.3), 21 (lineage 2.2.2), 9 (lineage 2.2.2), and 7 (lineage 4.4.1.1) participants. The smallest contains 2 participants.


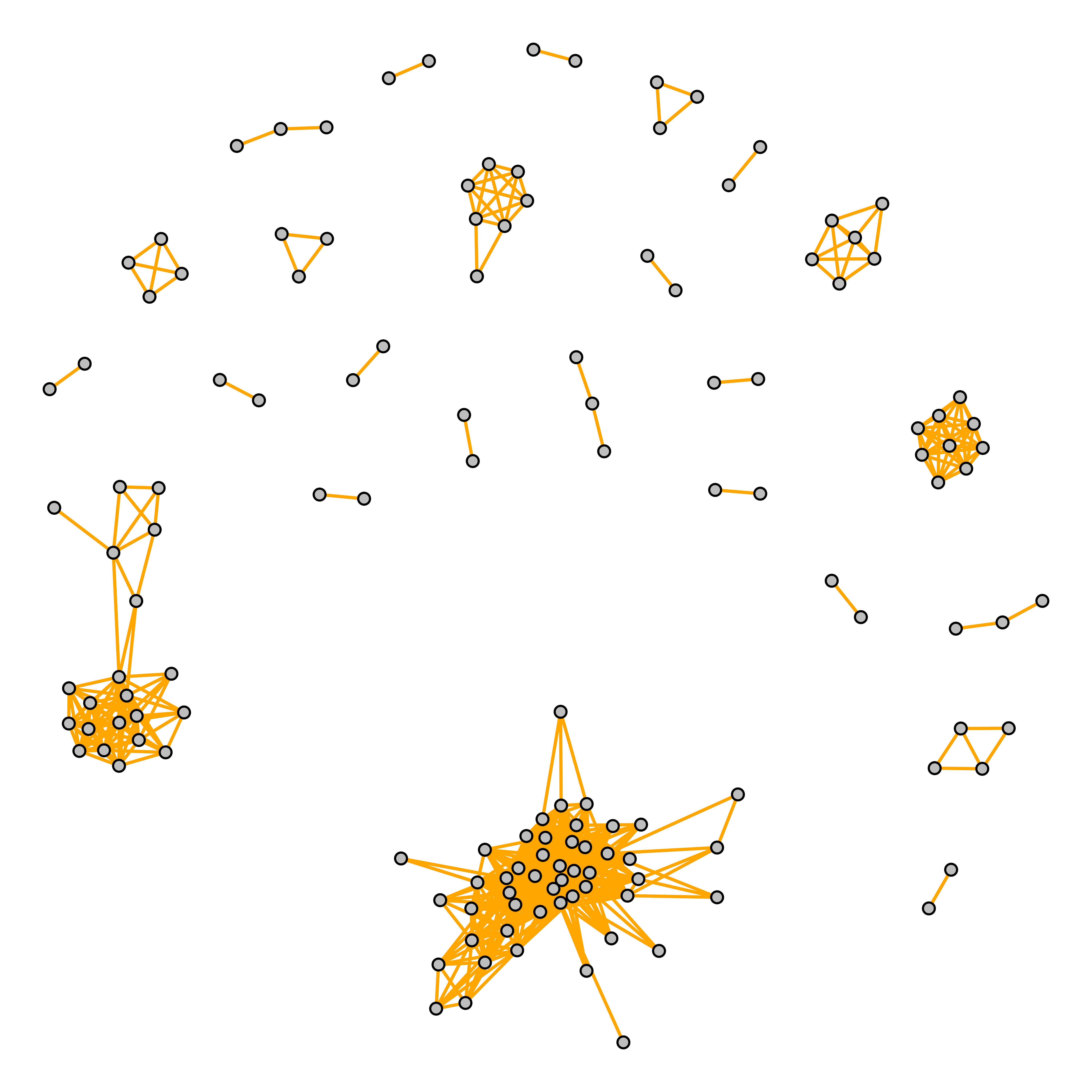


# Supplementary Tables

## Supplemental Table E1: Characteristics of enrolled study participants vs. non-enrolled persons with pre-XDR and XDR-TB

| **Characteristic** | **Enrolled cohort (n=305)** **N (%)** | **Not enrolled  (n=78)**  **N (%)** | **p-value** |
| --- | --- | --- | --- |
| Sex: Male | 168 (55) | 38 (49) | 0.31 |
| Female | 137 (45) | 40 (51) |  |
|  |  |  |  |
| Age, median (IQR) | 36 (30-44) | 35 (29-44) | 0.23 |
| Age categories, years:   0-12 | 1 (<1) | 3 (4) | 0.28 |
| 13-19 | 12 (4) | 4 (5) |  |
| 20-29 | 61 (20) | 18 (23) |  |
| 30-39 | 117 (38) | 27 (35) |  |
| 40-49 | 70 (23) | 15 (19) |  |
| 50-59 | 32 (10) | 8 (10) |  |
| ≥60 | 12 (4) | 3 (4) |  |
|  |  |  |  |
| Drug resistance: XDR TB | 100 (33) | 29 (37) | 0.47 |
| Pre-XDR (Fq resistant) | 130 (43) | 29 (37) |  |
| Pre-XDR (SLID resistant) | 40 (13) | 11 (14) |  |
| MDR+ other SL resistance | 9 (3) | 0 |  |
| SL resistance, not MDR | 26 (9) | 9 (12) |  |

IQR: Interquartile range; XDR TB: extensively drug-resistant tuberculosis; Fq: fluoroquinolone; SLID: second-line injectable drug; MDR: multidrug-resistant

## Supplemental Table E2: Locations of interactions with Close Contacts

| **Total Contacts** | **N=2,929** | |
| --- | --- | --- |
|  | **N** | **%** |
| **Location of Interaction** |  |  |
| Participant’s Home | 1402 | 48% |
| Friend, partner or family member’s home | 556 | 19% |
| Overnight visit location | 256 | 8.7% |
| Place of Entertainment (e.g., bar, restaurant) | 137 | 4.7% |
| Work or school | 130 | 4.4% |
| Shop, mall or market | 89 | 3.0% |
| Church, mosque or temple | 80 | 2.7% |

## Supplemental Table E3: Departments visited during outpatient clinic visits

| **Total outpatient clinics visited** | **N=668** | |
| --- | --- | --- |
|  | **N** | **%** |
| **Departments visited** |  |  |
| Tuberculosis | 451 | 68% |
| Pharmacy | 384 | 57% |
| Outpatient or urgent care | 301 | 45% |
| HIV | 292 | 44% |
| Radiology | 94 | 14% |
| Pre-natal or maternity care | 44 | 6.6% |
| Pediatrics | 16 | 2.4% |

## Supplemental Table E4: Sensitivity analysis of genotypic clustering and epidemiologic links using varying SNP thresholds.

| **SNP cutoff** | **Number of clusters** | **Clustered**  **N (%)** | **Range of participants per cluster** | **Median pairwise SNP difference** | **Participants with Close Contact links**  **N (%)** | **Participants with Casual Contact links**  **N (%)** | **Proportion of casual to close contact links** |
| --- | --- | --- | --- | --- | --- | --- | --- |
| 5 | 20 | 70 (28%) | 2-7 | 2 (IQR 1-4) | 4 (5.7%) | 23 (33%) | 5.75 |
| 7 | 25 | 97 (39%) | 2-15 | 4 (IQR 2-5) | 6 (6.2%) | 32 (33%) | 5.33 |
| 10 | 24 | 126 (50%) | 2-45 | 5 (IQR 2-7) | 11 (8.7%) | 50 (40%) | 4.55 |
| 12 | 25 | 141 (56%) | 2-49 | 6 (IQR 3-8) | 13 (9.2%) | 69 (49%) | 5.31 |
| 15 | 24 | 161 (64%) | 2-59 | 7 (IQR 3.5-10) | 19 (12%) | 78 (48%) | 4.11 |
| 20 | 24 | 185 (74%) | 2-63 | 7.5 (IQR 3.5-11) | 23 (12%) | 95 (51%) | 4.13 |

## Supplemental Table E5: SNP differences among pairs with differing types of epidemiologic links.

|  |  | Median (IQR) |
| --- | --- | --- |
| Close Contact | Direct person-to-person link | 7.5 (6-8) |
|  | Person-to-Person through a shared close contact | 7.5 (5-9) |
|  | Overlapping hospitalization | -- |
|  |  |  |
| Casual Contact | Residential Proximity | 8 (5.5-10) |
|  | Community Proximity | 8 (4-9) |
|  | Shared clinic | 8 (5-10) |
|  |  |  |
|  | No Epi Link | 10 (8-11) |

**References**

1. Van der Loo MP. The stringdist package for approximate string matching. 2014.

2. Wood DE, Salzberg SL. Kraken: ultrafast metagenomic sequence classification using exact alignments. *Genome biology* 2014; 15: R46.

3. Schmieder R, Edwards R. Quality control and preprocessing of metagenomic datasets. *Bioinformatics* 2011; 27: 863-864.

4. Li H, Durbin R. Fast and accurate short read alignment with Burrows-Wheeler transform. *Bioinformatics* 2009; 25: 1754-1760.

5. Eldholm V, Monteserin J, Rieux A, Lopez B, Sobkowiak B, Ritacco V, et al. Four decades of transmission of a multidrug-resistant Mycobacterium tuberculosis outbreak strain. *Nat Commun* 2015; 6: 7119.

6. Nguyen LT, Schmidt HA, von Haeseler A, Minh BQ. IQ-TREE: a fast and effective stochastic algorithm for estimating maximum-likelihood phylogenies. *Molecular biology and evolution* 2015; 32: 268-274.

7. Freschi L, Vargas R, Husain A, Kamal SMM, Skrahina A, Tahseen S, et al. Population structure, biogeography and transmissibility of Mycobacterium tuberculosis. *Nature Communications* 2021; 12: 6099.

8. World Health Organization. Catalogue of mutations in Mycobacterium tuberculosis complex and their association with drug resistance, second edition: World Health Organization; 2023.

9. Warren JL, Chitwood MH, Sobkowiak B, Colijn C, Cohen T. Spatial modeling of Mycobacterium tuberculosis transmission with dyadic genetic relatedness data. *Biometrics* 2023; 79: 3650-3663.
